# Comparative Biofilmomics of Antimicrobial-Resistant *Salmonella*: Serovar- and Host-Specific Signatures

**DOI:** 10.3390/ani16091302

**Published:** 2026-04-23

**Authors:** Lekshmi K. Edison, Subhashinie Kariyawasam

**Affiliations:** Department of Comparative, Diagnostic, and Population Medicine, College of Veterinary Medicine, University of Florida, Gainesville, FL 32610, USA; edison.le@ufl.edu

**Keywords:** *Salmonella enterica*, biofilm formation, antimicrobial resistance, multidrug resistance, serovar-specific adaptation, host adaptation, comparative biofilmomics, multi-omics integration, persistence, food safety

## Abstract

*Salmonella enterica* remains a major threat to animal and human health because of its broad host range, increasing antimicrobial resistance (AMR), and capacity to form biofilms. Biofilm formation enhances bacterial persistence in host tissues, farm environments, food-processing systems, and clinical reservoirs, while also contributing to their tolerance against antibiotics, disinfectants, and other stresses. However, biofilm capacity is not uniform across serovars and is influenced by host adaptation, niche specialization, and accessory genome content. This review synthesizes current knowledge on the relationship between biofilm formation, AMR, and serovar-specific adaptation in *Salmonella*. It examines biofilm-associated traits across various hosts (e.g., gastrointestinal tract and gallbladder, and environmental (e.g., food-production and clinical) niches, and discusses comparative evidence from genomic, transcriptomic, proteomic, and metabolomic studies. Particular attention is given to the emerging concept of comparative biofilmomics, which integrates phenotypic and multi-omics data across diverse serovars and host sources to identify conserved and niche-specific determinants of persistence. This framework may help define high-risk lineages that couple multidrug resistance (MDR) with enhanced biofilm-forming capacity. A better understanding of these linked traits will support the development of more targeted interventions for controlling persistent *Salmonella* in veterinary, food production, and public health settings.

## Simple Summary

*Salmonella* is a major cause of foodborne disease in humans and animals. Some strains are especially difficult to control because they can resist antibiotics and form biofilms, which are protective layers that help bacteria survive on tissues, farm surfaces, and food-processing equipment. However, not all *Salmonella* strains behave in the same way. Their ability to persist can vary by serovar, host source, and environment. This review analyzes how biofilm formation, antimicrobial resistance (AMR), and host adaptation are interconnected in *Salmonella*. It also introduces the concept of comparative biofilmomics, an approach that combines biofilm measurements with genome, gene-expression, protein, and metabolic data across different strains and host niches. By comparing these datasets together, researchers may better identify high-risk *Salmonella* lineages that combine strong persistence with drug resistance. This knowledge could inform improved *Salmonella* control strategies in animal production systems, food safety programs, and public health interventions.

## 1. Introduction

Antimicrobial-resistant (AMR) *Salmonella* represents a major global threat to both human and animal health [1]. As a leading cause of foodborne disease worldwide, *Salmonella enterica* is responsible for millions of infections annually, with significant morbidity associated with invasive and multidrug-resistant (MDR) lineages [2]. The increasing prevalence of extended-spectrum β-lactamase (ESBL)-producing and fluoroquinolone-resistant strains has further complicated treatment outcomes in both human and veterinary clinical settings [3]. Beyond classical resistance mechanisms, the ability of *Salmonella* to persist in diverse host and environmental niches is closely linked to its capacity to form biofilms, structured, surface-associated bacterial communities embedded within an extracellular polymeric substance (EPS) matrix [4].

Biofilms provide *Salmonella* with enhanced tolerance to antibiotics, disinfectants, desiccation, oxidative stress, and host immune defenses [5]. The biofilm lifecycle typically progresses through sequential stages: (i) reversible surface attachment, (ii) irreversible adhesion and microcolony formation, (iii) maturation into a three-dimensional architecture supported by curli fimbriae, cellulose, and other EPS components, and (iv) dispersion, enabling dissemination to new niches. Within biofilms, bacterial cells exhibit altered metabolic states, differential gene expression, and increased phenotypic heterogeneity, including the emergence of persister populations that survive antibiotic exposure [6]. These traits contribute to chronic carriage, environmental persistence, and recurrent contamination in food production systems [7].

Importantly, biofilm formation in *Salmonella* is not uniform across serovars [8]. The species encompasses over 2600 serovars with distinct host ranges, virulence strategies, and epidemiological patterns [9]. For example, broad-host-range serovars, such as *Salmonella enterica* serovar Typhimurium and serovar Enteritidis, commonly colonize poultry intestines and are associated with human gastroenteritis, whereas the host-adapted serovar *Salmonella enterica* serovar Dublin exhibits invasive disease phenotypes and long-term persistence in cattle [1]. Host-restricted serovars, including *Salmonella enterica* serovar Gallinarum, demonstrate genome degradation and niche specialization that may influence biofilm capacity and stress adaptation [10]. These serovar-specific differences extend beyond virulence to include variations in stress tolerance, plasmid carriage, and resistance gene distribution, all of which may shape biofilm behavior in host and environmental contexts. Table 1 summarizes the major *Salmonella* serovars and their associated hosts.

The intersection between AMR and biofilm biology is particularly critical. Biofilms not only reduce antibiotic penetration but also promote horizontal gene transfer through close cell-to-cell contact and increased stability of mobile genetic elements [6]. Plasmids carrying resistance determinants may co-localize with genes involved in adhesion, stress response, or surface colonization, potentially linking MDR phenotypes with enhanced persistence [11]. However, many studies have evaluated biofilm formation or AMR profiles independently, often using limited isolate panels and single phenotypic assays such as crystal violet staining. Few investigations systematically integrate serovar identity, host origin, antimicrobial phenotype, and multi-omics data within a unified comparative framework [12,13,14].

Understanding serovar- and host-specific biofilm differences in *Salmonella* is critical for explaining its ecological success and for guiding effective control strategies in food-processing and clinical settings. Although biofilm formation and AMR have been widely studied, a significant gap remains in integrating these traits across diverse serovars, host origins, and molecular backgrounds. This review addresses that gap by synthesizing current evidence on *Salmonella* biofilm biology, AMR, and serovar-specific adaptations from a comparative and integrative perspective. We evaluate key phenotypic and genotypic determinants of biofilm formation, examine the interplay between biofilm lifestyles and AMR mechanisms, and highlight how host adaptation and niche specialization shape persistence strategies. The emerging concept of “comparative biofilmomics” seeks to combine phenotypic biofilm assessment with genomic, transcriptomic, proteomic, and metabolomic analyses across multiple serovars and host niches. This integrative approach enables the identification of conserved versus serovar-specific biofilm determinants, the characterization of accessory genome contributions, and the definition of niche-adapted molecular signatures [15]. By overlaying AMR genotype and phenotype data with biofilm-associated pathways, comparative biofilmomics can reveal high-risk lineages that couple multidrug resistance with enhanced persistence potential [13]. By bridging phenotypic and genomic data, this review provides a framework for understanding the co-evolution of biofilm formation and AMR in *Salmonella* and for informing targeted intervention strategies in veterinary and public health contexts.

This review was conducted through a comprehensive search of the peer-reviewed literature using databases such as PubMed, Web of Science, and Google Scholar, focusing on keywords including “*Salmonella* biofilm”, “antimicrobial resistance”, “serovar-specific adaptation”, and “multi-omics”. Articles were selected based on their relevance to biofilm formation, AMR, host adaptation, and comparative omics in *Salmonella*.

## 2. Biofilm Adaptation Across Various Host and Environmental Niches

### 2.1. Gastrointestinal Tract—Primary Infection Site

In the intestinal lumen and mucosa, *Salmonella* infections typically manifest as acute gastroenteritis, but bacteria can also form surface-associated aggregates that resemble biofilm-like communities. The dominant enteric serovars associated with gastrointestinal colonization are *S.* Typhimurium and *S.* Enteritidis, which account for the majority of non-typhoidal salmonellosis cases in humans and animals. Within the intestinal niche, these serovars can attach to the mucus layer and epithelial surfaces, forming microcolonies that resemble early biofilm structures. Experimental studies in animal models have shown that *Salmonella* spp. are capable of forming biofilm-like communities within the intestine, suggesting that aggregation and matrix production contribute to persistence during infection [4,16,17]. Particularly in poultry, the cecum represents a major site of *Salmonella* persistence and colonization, where high bacterial loads can be recovered even when systemic infection is limited. Colonization of the cecal contents and epithelium contributes to prolonged intestinal carriage and fecal shedding, facilitating transmission to new hosts [17,18].

These intestinal biofilm-like structures likely facilitate bacterial survival in the gut environment, allowing organisms to persist despite host immune responses and enabling continued shedding in feces. In this context, extracellular matrix components such as curli fimbriae and cellulose contribute to bacterial aggregation and protection from host defenses. Although these structures are not always classical surface biofilms, they share several functional characteristics with biofilm communities, including enhanced stress tolerance and persistence within the host environment [19,20,21].

### 2.2. Gallbladder and Bile-Rich Environments—Chronically Persistence Niche

The gallbladder and biliary tract represent important niches for *Salmonella* persistence due to the high bile salt concentrations that shape bacterial physiology and gene expression. Chronic *Salmonella* carriers frequently harbor biofilms in this environment, most notably in infections caused by host-restricted *Salmonella enterica* serovar Typhi. In chronic typhoid carriers, more than 90% of individuals develop gallstones (cholesterol surfaces combined with bile), on which *S*. Typhi forms dense EPS-encased biofilms. These gallstone-associated biofilms facilitate long-term asymptomatic carriage and continuous fecal shedding, thereby contributing to transmission [22,23]. Experimental infection models further demonstrate that *Salmonella* can form microcolonies on the gallbladder epithelium, accompanied by epithelial invasion and aggregation on the mucosal surface [22]. These observations indicate that both cholesterol gallstones and epithelial surfaces within the biliary tract can support biofilm-like bacterial communities. Bile also plays a regulatory role in this process.

Studies have shown that bile salts strongly induce the expression of biofilm-associated regulators, including curli fimbriae genes (*csg* operons), with substantial increases in curli expression reported under bile-conditioned environments [23]. This bile-mediated induction likely enhances bacterial adhesion and promotes stabilization of biofilm structures within the gallbladder. Host-adapted invasive serovars appear to exploit this niche through distinct persistence strategies. While human-restricted serovar *S*. Typhi clearly utilizes gallstone-associated biofilms as a mechanism for chronic carriage [24], the cattle-adapted serovar *S*. Dublin, which can also cause invasive infections in humans [25], remains less well characterized in the hepatobiliary environment.

### 2.3. Food Production Environment (Poultry, Cattle, and Swine)—Transmission Niche

In agricultural and food-processing environments, *Salmonella* is frequently exposed to abiotic surfaces, low temperatures, and sanitation procedures that collectively influence biofilm development and persistence. Biofilm formation on food-contact materials such as polyvinyl chloride (PVC), stainless steel, nylon, and wood can support *Salmonella* biofilm formation, although surface properties, such as hydrophobicity and roughness, often enhance bacterial attachment. Rough or hydrophobic surfaces, such as nylon or unfinished wood, often promote thicker and more stable biofilms. Moreover, the physicochemical properties of biofilms can change over time, with mature biofilms becoming increasingly hydrophobic, which may further strengthen bacterial attachment and persistence on equipment surfaces [26]. Certain environmental conditions further influence biofilm development. Refrigeration temperatures commonly encountered in food storage do not prevent biofilm formation; in some cases, greater biofilm biomass has been reported at approximately 10 °C compared with 37 °C across multiple serovars [27]. Biofilm growth under such conditions is particularly relevant to cold-chain environments, where persistent contamination may occur during processing and storage. In addition, biofilm-associated cells exhibit increased tolerance to environmental stresses, including desiccation and exposure to sanitizing agents, largely due to the protective effects of the EPS matrix. These observations are consistent with pre-harvest studies demonstrating that *Salmonella* can persist in poultry production systems and contaminate the environment even in the absence of clinical disease [28].

### 2.4. Clinical and Environmental Reservoirs—Secondary Reservoir Niche

Beyond animal hosts and agricultural settings, *Salmonella* can persist in a range of human-associated and natural environments through biofilm formation. In clinical and community settings, *Salmonella* has been recovered from biofilms associated with plumbing systems, sink drains, pipes, and, more occasionally, medical devices or other built-environment surfaces, where biofilm growth enhances persistence and reduces susceptibility to cleaning and disinfection [29]. Similarly, wastewater systems and natural aquatic environments may serve as reservoirs for *Salmonella* within mixed-species biofilms, thereby contributing to environmental maintenance and potential transmission [30,31].

In these non-host niches, biofilm formation is closely intertwined with AMR. Many environmental and clinical *Salmonella* isolates carry MDR plasmids, and biofilm growth can favor both plasmid maintenance and continued horizontal transfer. Biofilms create spatially structured communities with nutrient and antimicrobial gradients, allowing subpopulations to persist under reduced selective pressure while retaining mobile resistance elements [32]. Experimental studies have shown that biofilm-grown bacteria can preserve MDR plasmids and maintain their transmissibility for longer periods than comparable planktonic populations, even in the absence of direct antibiotic selection [30]. Consequently, *Salmonella* biofilms in environmental settings may function as reservoirs of both infection and resistance genes [33,34]. Once established on abiotic surfaces, these communities can support long-term bacterial survival, complicate mitigation efforts, and facilitate the continued circulation of MDR lineages [33,35]. These observations underscore the importance of control strategies that target not only planktonic cells but also the biofilm structures that sustain persistence and resistance in clinical and environmental reservoirs.

## 3. Serovar Diversity in Biofilm Formation and Antimicrobial Resistance

### 3.1. Classical Phenotype Comparisons

Biofilm formation in *Salmonella enterica* varies substantially across serovars and environmental conditions. Early mechanistic studies established that the RDAR (red, dry, and rough) morphotype, which is regulated primarily by *csgD* and associated with curli and cellulose production, underlies robust surface biofilm formation in many non-typhoidal serovars. However, expression of RDAR morphology is temperature-dependent, typically enhanced at 22–28 °C and reduced at 37 °C, highlighting environmental regulation of biofilm phenotypes [36].

Comparative phenotypic studies have demonstrated serovar-specific differences in biofilm formation on abiotic surfaces, including polystyrene, stainless steel, and glass. For example, an experiment involving poultry-associated isolates showed that *Salmonella enterica* serovar Schwarzengrund frequently forms strong biofilms at both room temperature and during refrigeration, whereas serovars Heidelberg and Newport tend to form weaker biofilms under similar laboratory conditions. Surface type also influences biofilm formation, especially stainless steel and plastic surfaces commonly used in food-processing environments exhibit differential attachment efficiencies depending on the serovar and strain background [37].

Within serovars, lineage-specific differences further contribute to phenotypic heterogeneity. In *S.* Typhimurium, sequence type (ST) 19 strains, globally distributed and associated with gastroenteritis, typically display classical RDAR morphology and environmental persistence. In contrast, the invasive African lineage ST313 shows altered metabolic profiles, genome degradation, and reduced biofilm formation under certain laboratory conditions, consistent with niche adaptation toward systemic infection rather than environmental survival. These differences suggest that long-term survival and biofilm capacity may reflect evolutionary trade-offs between environmental persistence and host adaptation [38]. Several studies have systematically compared biofilm-forming capacity among *Salmonella* serovars under different environmental conditions. Representative investigations highlighting serovar- and lineage-specific biofilm phenotypes are summarized in Table 2. Collectively, these studies demonstrate substantial heterogeneity in biofilm-forming capacity across *Salmonella* serovars and lineages. Environmental conditions such as surface type, temperature, and stress exposure further modulate biofilm phenotypes, suggesting that persistence traits are shaped by both genetic background and ecological context [36].

### 3.2. Link to Antimicrobial Resistance

The relationship between *Salmonella* serovar designation and AMR pattern is well established. Certain serovars are closely associated with MDR and ESBL production. Reports have highlighted the emergence of *Salmonella enterica* serovar Mikawasima in Europe, including outbreaks and increasing detection in humans and food sources, with some isolates exhibiting AMR traits, including β-lactamase-associated resistance [44,45]. *Salmonella enterica* serovar Kentucky, particularly ST198, has emerged globally as a fluoroquinolone-resistant and MDR lineage frequently isolated from poultry and humans [46]. The serovar Heidelberg has been associated with plasmid-mediated extended-spectrum cephalosporin resistance determinants (e.g., *bla*_CMY-2_) in North America, contributing to reduced susceptibility to third-generation cephalosporins [47,48]. Similarly, serovar Dublin, a cattle-adapted serovar associated with invasive disease, frequently carries MDR plasmids and resistance determinants linked to livestock antibiotic exposure [25]. These serovars demonstrate how host-adapted or production-associated lineages can accumulate resistance determinants through clonal expansion and mobilization of mobile genetic elements.

The intersection between AMR and biofilm formation, however, remains complex. Within biofilm communities, AMR can be influenced through both plasmid-mediated mechanisms and chromosomal mutations. Biofilms provide a dense and structured environment that facilitates horizontal gene transfer, particularly conjugation, thereby enhancing the dissemination and stability of plasmids carrying resistance determinants. In addition, several plasmids encode both AMR genes and adhesion-related factors, which may further stabilize these elements within biofilm-associated populations [49,50]. At the same time, biofilm-associated stress conditions, including oxidative stress, nutrient limitation, and antimicrobial exposure, can promote the selection of chromosomal mutations associated with resistance [51]. Furthermore, reduced metabolic activity and the presence of persister cells within biofilms contribute to increased tolerance to antibiotics, even in the absence of specific resistance genes [6,52]. Together, these mechanisms highlight the role of biofilms as critical environments that support both the acquisition and maintenance of AMR.

Biofilms inherently confer increased tolerance to antimicrobials due to restricted penetration, altered metabolic states, and stress-response activation [53]. Several studies report that strong biofilm-forming isolates often exhibit higher MDR rates than weak biofilm producers. For example, poultry-associated isolates exhibiting robust biofilm phenotypes were more likely to harbor resistance to multiple antibiotic classes [6,42]. Conversely, some studies have reported no consistent correlation between planktonic AMR phenotype and biofilm biomass as measured by crystal violet assays. This inconsistency suggests that resistance genes and biofilm determinants may not be directly linked genetically, but rather co-selected under shared environmental pressures such as disinfectant exposure, metal stress, or antibiotic use [54]. Furthermore, plasmids carrying AMR determinants can also encode adhesion factors or otherwise alter colonization phenotypes (e.g., via conjugative pili), potentially stabilizing resistance within biofilm communities [55]. Overall, while certain high-risk serovars combine MDR with persistence traits, the relationship between AMR genotype and biofilm phenotype remains context-dependent and incompletely resolved.

## 4. Genomic and Pangenomic Basis of Biofilm Traits

### 4.1. Core vs. Accessory Biofilm Genes

Most *Salmonella* serovars share a conserved set of “core” biofilm genes. These include the curli fimbriae operons (*csgBAC* and *csgDEFG*), the cellulose synthase genes (*bcs* operon), the diguanylate cyclase *adrA*, and global regulators such as *csgD* and *rpoS*. In broad-host-range serovars such as *S*. Typhimurium and *S*. Enteritidis, these loci are intact and drive the classic RDAR (curli/cellulose) biofilm phenotype. By contrast, host-restricted serovars often accumulate mutations or pseudogenes in these pathways. For example, human-specific *S*. Typhi carries a premature stop codon in *csgD*, which leads to truncating the CsgD regulator and abolishing curli/cellulose expression [56]. Consequently, broad-host-range serovars such as Typhimurium and Enteritidis generally retain intact *csg* and *bcs* loci along with regulators like *adrA*, *csgD*, and *rpoS*, enabling strong biofilm formation [56], whereas host-restricted serovars, including Typhi, Paratyphi, Gallinarum, and Pullorum, often show inactivation or degradation of these genes, which correlates with reduced biofilm capacity and altered colony phenotypes [57]. Thus, core biofilm machinery is part of the *Salmonella* pangenome, but its functionality can be lost in specialized lineages that no longer require environmental persistence.

In addition to these core elements, *Salmonella* genomes carry various accessory genes that modulate biofilm phenotypes. For example, *bapA* (biofilm-associated protein A) encodes a large, secreted adhesin that is co-regulated with curli/cellulose by *csgD* and contributes to pellicle formation [19]. The presence or absence of *bapA* and other adhesin genes, such as genes for long polar fimbriae, type 1 fimbriae, or sigma-dependent fimbriae, varies among serovars [58]. Some plasmids and genomic islands also encode extracellular polysaccharide biosynthesis or secretion systems that can enhance biofilm structure. For instance, certain IncF or IncI plasmids in typhoidal strains carry additional adhesion factors and toxin-antitoxin modules that affect surface attachment [59]. Table 3 summarizes representative genomic and expression differences in biofilm-associated genes across serovars with different host ranges. In summary, while the core regulators and matrix genes are conserved, an accessory “biofilm toolkit”, consisting of additional adhesins, secretion factors, and surface polysaccharide modifiers, can provide serovar-specific enhancements to biofilm formation in particular niches.

### 4.2. Mobile Genetic Elements as Genomic “Bridges” Between Biofilm Ecology and Antimicrobial Resistance

Mobile genetic elements (MGEs) frequently harbor genes affecting both AMR and biofilm-related adhesins, thereby coupling AMR and persistence. For example, the emergent *Salmonella enterica* serovar Infantis megaplasmid pESI carries multiple AMR determinants and two novel chaperone-usher fimbrial clusters (*ipf* and *klf*). These plasmid-encoded fimbriae contribute to host colonization while the same plasmid confers AMR, thereby coupling virulence with resistance [76]. Likewise, the virulence plasmid of *S*. Typhimurium (IncF-type) encodes genes for plasmid-encoded fimbriae (*pef*), linking adhesion to plasmid-borne virulence factors [77]. Additionally, many conjugative plasmids, such as IncF, IncI, and IncQ, are known to harbor AMR gene cassettes alongside virulence/adhesion genes (fimbriae or toxins) [78].

In addition to plasmids, class-1 integrons in *Salmonella* capture multiple resistance cassettes; while not classically “biofilm” genes, they often co-exist with stress-response or efflux genes that aid survival under antimicrobial and environmental stress [79]. Several *Salmonella* prophages carry accessory genes (e.g., *sodC* superoxide dismutase and *yidE*) that enhance oxidative stress tolerance or adhesion. Although the mechanisms underlying biofilm formation are not fully defined, prophage-encoded factors could modulate stress resilience or cell-surface properties [80,81]. Overall, horizontally transferred elements can create genotypes where AMR and attachment capabilities are inherited together, potentially selecting for biofilm-competent, drug-resistant strains.

### 4.3. Comparative Genomics and Pangenome Analyses

Large-scale genomic comparisons of *Salmonella* serovars reveal consistent patterns in gene content that correlate with biofilm capacity and host niche. Pangenome analyses reveal a core genome shared by biofilms and a large accessory genome comprising genes variably distributed among serovars. For instance, genomic studies showed that the cattle-adapted serovar *S*. Dublin carries more mutations/pseudogenes in metabolic pathways, whereas the broad-host serovar *S*. Enteritidis retains intact anaerobic metabolism genes [82]. Proteomic profiling under gut-mimicking conditions confirmed that Dublin expressed many stress-response genes and virulence-associate genes, while Enteritidis overproduced anaerobic metabolic enzymes [83]. Together, these findings suggest that *S*. Enteritidis appears adapted for fermentative growth within the intestinal biofilm niche, whereas *S*. Dublin emphasizes stress defense mechanisms consistent with invasive disease. Similar comparative genomic analyses in *S*. Enteritidis have identified host-specific virulence and adaptation factors [84].

A pan-Genome-Wide Association Study (pan-GWAS) of 78 strains (21 *S*. Typhimurium, 57 monophasic variant) identified clear deletions in the monophasic clones associated with reduced adhesion potential. Notably, the entire fimbrial operon *stbABCDE*, which encode a major adherence factor, is deleted in the monophasic clones, and portions of the *iroA* salmochelin (siderophore) locus are also missing. The siderophore receptor *iroN* was strongly associated with higher biofilm production in *S*. Typhimurium [85]. Thus, two very close lineages differ in adhesion capacity: the ancestral Typhimurium retains the full fimbrial assembly for attachment, whereas the monophasic variant has lost it, predicting diminished biofilm formation. Genomic comparisons of poultry-restricted *S*. Gallinarum and *S*. Pullorum show extensive pseudogenization (accumulation of non-functional pseudogenes) of ~25% of genes relative to Enteritidis, implying loss of unnecessary functions in the chicken host. Many of these degraded genes are likely involved in environmental survival, such as surface structures and transporters [57]. Additionally, a proteomic study identified serovar-specific differences in proteins such as β-lactamases and O-antigen modification enzymes and confirmed that Gallinarum and Pullorum have lost numerous accessory functions [86]. This suggests that these host-restricted serovars depend on a narrower repertoire of virulence mechanisms (e.g., *Salmonella* Pathogenicity Island [SPI]-mediated invasion) rather than maintaining the broader environmental persistence traits, including biofilm formation, observed in generalist serovars.

Altogether, pangenome comparisons show that broad-host serovars (e.g., Typhimurium, Enteritidis) generally carry a full complement of core and accessory biofilm genes, whereas host-adapted serovars (e.g., Dublin, Gallinarum, Pullorum) often lose or downregulate these genes. These genomic signatures correspond with observed biofilm phenotypes: the human-restricted Typhi and poultry-restricted Gallinarum are weak biofilm formers, while Typhimurium and Enteritidis form strong biofilms. Even without direct biofilm assays, the genomic and pangenomic patterns, together with other ‘omics’, can provide insight into the ecological niches preferred by different serovars. For example, elevated anaerobic metabolism in Enteritidis implies it is well-adapted to low-oxygen intestinal biofilm environments, whereas Dublin and Gallinarum show genomic signatures consistent with prioritizing acute virulence over persistence [10,83].

## 5. Transcriptomic, Proteomic and Metabolomic Signatures of Biofilm in Different Serovars and Hosts

### 5.1. In Vitro Biofilm vs. Planktonic Omics

Multiple studies have compared *Salmonella* gene and protein expressions in biofilms versus planktonic cultures. In general, biofilm-grown cells upregulate surface attachment and matrix pathways and downregulate motility. For example, the curli operon (*csgBACDEFG*) and cellulose and colanic acid biosynthesis genes (key components of the EPS matrix) are strongly induced in biofilms, whereas flagellar, type-1 fimbriae (*fim* operon) and SPI-2 (type III secretion) genes are often downregulated in mature biofilms. Key stress-response and regulatory factors, such as RpoS-regulated stress chaperones (e.g., Dps, OsmY, BtuE) and oxidative-defense proteins, are elevated in biofilms, indicating a sensitive stationary phase and protection mode [23,87]. Proteomic analysis confirmed this trend, showing that biofilm cells upregulate stress-protective proteins, including Dps, OsmY, TrxA, and SspA, and DNA repair proteins, collectively increasing resistance to oxidative, osmotic, and nutritional stresses within the biofilm microenvironment [88].

Metabolic pathways are also reprogrammed during biofilm growth. Genes involved in tryptophan, purine biosynthesis and propanediol utilization are often upregulated in bacteria in biofilms, reflecting some core respiratory functions that adjust to low-nutrient conditions [23,89]. In parallel, genes linked to membrane integrity and efflux systems, including bile-stress regulators such as *marA*, components of the *tol* operon, and multidrug efflux pumps (e.g., EmrAB or Mdt family transporters), are frequently elevated during biofilm development, likely reflecting increased exposure to environmental and antimicrobial stresses [23,90]. Notably, omics data stratified by serovar or AMR phenotype remain limited. One transcriptomic study reported that a strong biofilm-forming *Salmonella* Typhimurium strain upregulated the multidrug efflux encoding *mdtL* [90], suggesting a potential link between biofilm propensity and AMR mechanisms. However, most omics investigations have been conducted using a single reference strain, and comprehensive comparative analyses across multiple serovars or resistance phenotypes are still lacking.

### 5.2. Serovar-Specific Omics Under Host-Mimicking Conditions

Some proteomic and transcriptomic studies have compared different *Salmonella* serovars under conditions simulating host environments. In one such study, *S*. Enteritidis and host-adapted *S*. Dublin were grown in gut-like media. Dublin enriched stress-response and virulence proteins (e.g., stress chaperones, T3SS effectors), whereas Enteritidis showed higher levels of enzymes involved in anaerobic metabolism [83]. This suggests Dublin is tuned to survive harsh inflammatory conditions, while Enteritidis emphasizes fermentative growth in the gut. Such metabolic signatures could influence biofilm traits: e.g., higher anaerobic metabolism in Enteritidis might correlate with biofilms optimized for low-oxygen niches. Other serovar comparisons similarly reveal metabolic and virulence differences but occasionally highlight classic adhesion factors. For example, proteomic profiling of *S*. Gallinarum vs. *S*. Enteritidis, both isolated from poultry, found differential expression of enzymes involved in energy production and nucleotide metabolism, as well as several virulence-associated proteins, including SPI-1 (*Salmonella* Pathogenicity Island-1) effectors, RfbS and Hsp90, but did not prominently identify known biofilm matrix components [91].

Similarly, studies comparing *S*. Typhimurium, Pullorum, Gallinarum and Enteritidis have identified serovar-specific metabolic pathways, such as cysteine/sulfate metabolism, and regulatory proteins, underscoring broad physiological differences. In these cross-serovar omics, surface or adhesion proteins were not a major focus, suggesting that host adaptation may involve deeper metabolic rewiring [86,92]. Overall, these results imply that host-specific niches drive serovar differences, especially in stress tolerance and anaerobic growth, which, in turn, could shape each serovar’s propensity to form specific biofilm phenotypes; however, more targeted studies are needed to link specific biofilm adhesins.

### 5.3. Host-Specific Omics and Salmonella Biofilm Responses

Host transcriptomic and proteomic responses to *Salmonella* infection can shed light on the environmental pressures bacteria face in vivo. For example, transcriptomic analysis of chicken cecum infected with *S*. Enteritidis reveals a strong inflammatory signature, with marked upregulation of cytokine and innate immune genes and enrichment of immune pathways such as Toll-like receptor (TLR) signaling pathway, by 3 days post-infection, whereas by 14 days post-infection, host metabolic processes become more prominent as the chicken adapts to infection [93]. Another integrative chicken cecum omics analysis also found that Enteritidis induces immune gene expression while suppressing host metabolic enzymes [94]. These opposing shifts, such as immune activation and metabolic downregulation, suggest that infection drives host inflammation. Such niche conditions, characterized by elevated cytokines and reactive oxygen and nitrogen species, may favor biofilm-associated traits. Chronic inflammation can release nutrients (iron and amino acids) and cause tissue damage, creating an environment that supports bacterial persistence and biofilm formation [4].

Host niche factors beyond inflammation also modulate biofilms. Bile in the gallbladder acts as a strong environmental cue. In simulated gallstone biofilms, *S*. Typhimurium markedly induces curli production. Under these conditions, *csgA* and *csgB* transcripts increase more than 80-fold, whereas fimbrial genes (*fim*) and SPI-2 (*Salmonella* Pathogenicity Island-2) genes are strongly downregulated. This pattern suggests that bile selectively promotes adhesive and curli-rich biofilms that may facilitate bacterial colonization of gallstone colonization while suppressing invasive traits [23,95]. Although data on host mucus signals remain limited, mucus glycans and spatial heterogeneity in the intestinal environment may also shape *Salmonella* attachment and matrix production [96]. Overall, reciprocal host–pathogen interactions indicate that host immunity (e.g., inflammation and reactive oxygen species) and niche factors (e.g., bile, oxygen gradients, and mucus) exert selective pressures that promote distinct *Salmonella* biofilm lifestyles in different tissues.

### 5.4. Metabolomic Signatures of Biofilm Formation in Salmonella

Metabolomic analyses are beginning to reveal the metabolic reprogramming underlying *Salmonella* biofilms. In *S*. Enteritidis, an LC-MS study identified approximately 120 metabolites that differed between planktonic and sessile cells. Planktonic populations were enriched in amino acid precursors and polyamines, including proline, phenylalanine, putrescine, and cadaverine, reflecting active growth and stress adaptation. In contrast, biofilm-associated bacteria accumulated nucleotides and related metabolites, including lysine, adenosine, purines, pyrimidines, and citrate, which were particularly abundant, suggesting enhanced nucleotide biosynthesis and maintenance of redox balance. Pathway enrichment analysis further indicated that purine and pyrimidine metabolism, arginine-proline metabolism, and vitamin B6 metabolism were significantly altered in biofilm cells [97]. Together, these trends suggest that biofilm-associated Enteritidis prioritize energy storage and anabolic precursors that support matrix production and stress survival, whereas planktonic bacteria allocate resources to rapid-growth intermediates.

Environmental constraints such as oxygen limitation and host-derived nutrients likely shape the metabolic patterns observed in *Salmonella* biofilms. For example, increased arginine metabolism may help buffer acidic or inflammatory stress, while elevated nucleotide pools may support DNA repair and maintenance during the stationary-phase conditions typical of biofilms. Differences among serovars are also plausible. However, only a few studies have examined biofilm metabolomes across multiple serovars [97]. Future comparative metabolomic analyses will therefore be important for understanding how metabolic rewiring contributes to the distinct biofilm phenotypes observed among *Salmonella* lineages.

## 6. Methodological and Analytical Framework for Comparative Biofilmomics

### 6.1. Ideal Experimental Framework

**Figure 1:**
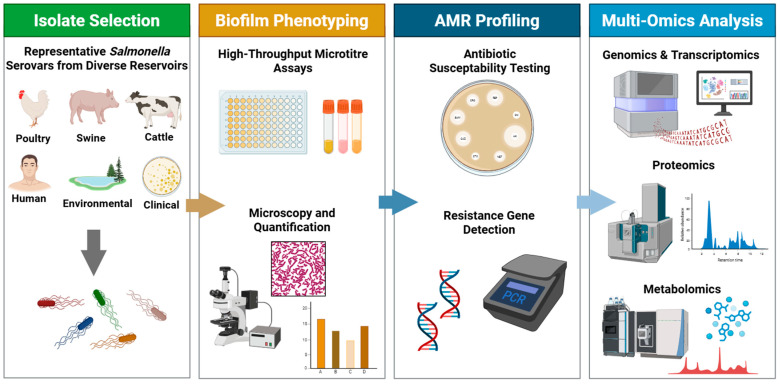
Comparative biofilmomics workflow for the integrated analysis of biofilm formation in *Salmonella*.

A comparative biofilmomics approach requires a systematic, high-throughput collection of phenotypic and multi-omics data across diverse pathogens. Figure 1 presents a proposed comparative biofilmomics workflow integrating isolate selection, biofilm phenotyping, AMR profiling, and multi-omics analyses. This framework should be built around a standardized isolate panel containing multiple serovars and host sources. For example, such a panel includes 10–20 isolates from each of 4–5 important *Salmonella* serovars, such as Typhimurium, Enteritidis, Heidelberg, I 4,[5],12:i:-, and Infantis, obtained from diverse reservoirs including humans, poultry, swine, cattle, and the environment [98,99]. Such diversity is essential because biofilm formation is highly strain- or serovar-specific and strongly influenced by environmental context. In *Salmonella*, variation is evident even at the species level [100]. Sarjit et al. reported that strains belonging to the species *enterica* were generally weaker biofilm formers than non-*enterica* species, such as *bongori* and subspecies *arizonae* and *diarizonae*. These phenotypic differences are linked to the presence or absence of key biofilm-associated genes [98]. Therefore, an ideal isolate panel should include not only the major epidemiologically relevant serovars but also sufficient phylogenetic and ecological diversity to support meaningful comparative analyses.

Each isolate within the panel should be subjected to parallel phenotypic and multi-omic analyses under standardized experimental conditions. For biofilm phenotyping, high-throughput microtiter plate assays, such as crystal violet staining, and/or automated microscopy-based approaches may be used to quantify initial attachment and total biofilm biomass. Experimental conditions, including surface material, temperature, nutrient availability, and exposure to stressors, should be selected to reflect biologically and industrially relevant niches, such as stainless steel or meat-associated surfaces under ambient or refrigeration temperatures [99,101]. In parallel, antimicrobial susceptibility testing, including minimum inhibitory concentration (MIC) profiling, should be performed to characterize AMR phenotypes. At the same time, each isolate should undergo whole-genome sequencing (WGS), ideally using long-read platforms (PacBio/Oxford Nanopore Technology/SMRT sequencing) combined with Illumina polishing, to support robust comparative genomic analyses. Under both biofilm-inducing and control conditions, transcriptomic profiling by RNA sequencing should be conducted to identify differentially expressed genes, while quantitative proteomic analyses, such as LC–MS/MS, can be used to measure changes in protein abundance. Where feasible, metabolomic profiling may also be incorporated to identify biofilm-associated metabolic signatures. Generating all of these datasets within a unified experimental framework would enable direct integration of phenotypic, genomic, and functional data, thereby facilitating the identification of molecular determinants associated with biofilm formation.

Importantly, experimental design must prioritize comparability across all bacterial isolate groups. Each serovar–host subset should include multiple isolates to account for within-serovar variability, and all samples should be processed using identical protocols, including the same media, incubation conditions, and sampling time points. The incorporation of automation and robotic platforms, such as liquid handling systems and plate readers for biofilm assays, would further enhance throughput, standardization, and reproducibility. Conceptually, this approach aligns with the “omics” framework proposed by Azevedo et al., who introduced the term “biofomics” to describe the generation of large, standardized biofilm datasets for the identification of biologically meaningful signatures [101]. In practice, this strategy resembles established large-scale pathogen surveillance platforms, particularly those used in AMR monitoring, but extends them through the integration of targeted biofilm phenotyping.

### 6.2. Integrated Bioinformatics and Machine Learning

The data generated through the above framework must be integrated using advanced bioinformatic approaches. Initially, dedicated analytical pipelines should be applied to each data type independently. Whole genomes should be assembled, annotated, and analyzed through pangenome-based comparisons. Transcriptomic data should be assessed for differential expression, and proteomic spectra should be quantified, and functionally annotated. At each stage, rigorous quality control, normalization, and batch-correction procedures are essential to ensure data reliability and cross-platform comparability. Subsequent analyses should focus on multi-omics integration. Two broad strategies are common: horizontal integration, in which each omics layer is analyzed separately and the resulting signals are compared or intersected, and vertical integration, in which multiple omics layers are combined directly at the feature level [102].

For example, transcripts (transcriptomics) identified as differentially expressed genes in biofilms versus planktonic conditions can be cross-checked with proteins that accumulate in biofilms (proteomics), and both can be mapped onto genetic presence/absence patterns across isolates (genomics). In addition, network-based approaches, including gene co-expression networks, protein–protein interaction maps, and metabolic pathways reconstruction, can facilitate the identification of biologically meaningful links across omics layers. Recent studies have emphasized that such integrative analyses can uncover composite biomarkers and regulatory signatures that would remain undetected when each molecular layer is examined in isolation [14,102,103].

Machine learning (ML) provides a powerful approach for defining biofilm signatures from these datasets [104,105]. Unsupervised methods, such as clustering and dimensionality reduction, may cluster isolates according to biofilm phenotype and reveal patterns across omics layers, whereas supervised methods can be used to predict biofilm-forming capacity or MDR status from omics-derived features [104]. Feature selection algorithms, including random forests with embedded importance scoring, LASSO regression, and more recent approaches such as SHAP-based interpretation, can help identify the most informative genes or proteins [106]. For example, cancer research has demonstrated that ML can integrate multi-omics data through feature selection to identify key molecular markers, and similar strategies could be applied in this context [102,107]. Models could be trained to classify isolates as high- or low-biofilm formers or MDR or susceptible, based on their multi-omics profiles. Recursive feature elimination or penalized regression may further refine a compact set of markers, including genes, transcripts, and proteins, that together define a biofilm signature [106]. More advanced approaches, such as deep learning or ensemble methods, may also be applicable, as has been shown for AMR prediction in *Salmonella* [108], provided that careful cross-validation is used to minimize overfitting, which remains a common challenge.

A promising extension of this framework would be the application of multi-task learning, in which each host source or serovar is treated as a related task within a shared predictive model. For example, a multi-task neural network could simultaneously predict biofilm-forming capacity in chicken, swine, and human isolates by sharing core model parameters while allowing task-specific variation. Such an approach could help identify genes whose association with biofilm formation is conserved across hosts, as well as those that are context-dependent. Although direct applications in comparative biofilmomics remain limited, multi-task learning has been successfully used in other heterogeneous multi-omics settings, including single-cell multi-omics integration [109]. In practice, each isolate could be annotated not only with biofilm phenotype data but also with relevant covariates, such as serovar, host source, and environmental condition, enabling multi-task or hierarchical models to distinguish broadly conserved biofilm determinants from niche-specific regulators.

At the same time, the results of ML analyses must be biologically meaningful and not based only on statistical patterns. As highlighted by Sampathkumar et al. (2026), biofilm formation is already known to involve multiple genetic determinants, including capsular polysaccharide loci, fimbriae, quorum-sensing regulators, and efflux pumps; thus, integrative analyses should both validate established mechanisms and identify novel contributors [110]. A useful precedent is provided by *Klebsiella pneumoniae*, in which Li et al. (2024) integrated genomic, transcriptomic, and phenotypic data to show that biofilm formation co-occurs with MDR genes and is influenced by several regulatory systems, including capsule synthesis, fimbriae, quorum sensing, and efflux pathways [110,111]. A similar multi-layered framework in *Salmonella* or other bacterial pathogens could reveal both well-recognized biofilm regulators, such as CsgD and cellulose synthase components, and previously unrecognized markers associated with high-risk biofilm phenotypes.

## 7. Future Directions

Despite significant advances, important gaps remain in our understanding of *Salmonella* biofilm biology. Most omics studies have focused on single strains under simplified laboratory conditions, and systematic cross-serovar biofilmomics analyses are still lacking. Comparative studies incorporating multiple serovars with different host ranges, ecological niches, and AMR profiles will be essential to distinguish conserved biofilm mechanisms from lineage-specific adaptations. Another major limitation is the scarcity of host-matched datasets. Many experiments do not replicate the complex environmental signals encountered during infection, such as bile exposure, mucus glycans, immune mediators, and oxygen gradients. Future studies should therefore examine biofilm formation in physiologically relevant systems, including poultry or bovine intestinal models, gallbladder environments, and organoid-based infection platforms.

Several regulatory layers also remain underexplored. Emerging evidence suggests that epigenetic regulation, small RNAs (sRNAs), noncoding RNAs, and extracellular vesicles may influence biofilm development and bacterial persistence; however, these mechanisms have received limited attention in *Salmonella*. Integrating these regulatory elements with genomic, transcriptomic, proteomic, and metabolomic data will provide a more comprehensive understanding of biofilm regulation. Finally, progress in this field would benefit from collaborative biofilmomics frameworks and shared databases that enable researchers to deposit standardized omics and phenotypic biofilm data across strains and experimental conditions. Such integrative approaches will help identify high-risk lineages that combine strong biofilm capacity with AMR and will ultimately support the development of targeted strategies to control *Salmonella* persistence in both veterinary and public health contexts.

## Figures and Tables

**Table 1:** Host association categories of selected *Salmonella* serovars (broad host range, host-adapted, and host-restricted).

*Salmonella* Serovar *	Group (O Antigen)	Host Association
Typhimurium	B (O:4)	Broad-host-range
Enteritidis	D1 (O:9)	Broad-host-range
Heidelberg	B (O:4)	Broad-host-range
Kentucky	C2-C3 (O:8)	Broad-host-range
Infantis	C1 (O:6,7)	Broad-host-range
Newport	C2-C3 (O:8)	Broad-host-range
Dublin	D1 (O:9)	Cattle-adapted, occasionally humans
Gallinarum	D1 (O:9)	Poultry-restricted
Pullorum	D1 (O:9)	Poultry-restricted
Bredeney	B (O:4)	Poultry-restricted
Typhi	D1 (O:9)	Human-restricted
Paratyphi A	A (O:2)	Human-restricted
Monophasic 4,[5],12:i:-	B (O:4)	Broad-host-range
Hadar	C2-C3 (O:8)	Broad-host-range
Schwarzengrund	B (O:4)	Broad-host-range
Thompson	C1 (O:6,7)	Broad-host-range
Agona	B (O:4)	Broad-host-range
Brandenburg	B (O:4)	Broad-host-range
Montevideo	C1 (O:6,7)	Broad-host-range
Senftenberg	E (O:3,10)	Broad-host-range
Derby	B (O:4)	Broad-host-range
Braenderup	C1 (O:6,7)	Broad-host range
Muenchen	C2-C3 (O:8)	Broad-host-range
Lagos	B (O:4)	Broad-host-range
Labadi	C2-C3 (O:8)	Broad-host-range
Anatum	E (O:3,10)	Broad-host-range
Give	E (O:3,10)	Broad-host-range
Mbandaka	Group C1 (O:6,7)	Broad-host-range
Mikawasima	Group C1 (O:6,7)	Broad-host-range
Manhattan	C2-C3 (O:8)	Broad-host-range
Cerro	K (O:18)	Cattle-adapted, swine-associated

* Data adapted from the *Salmonella* Serovar Wiki (Cornell Food Safety Laboratory). Available at: https://fsl-mqip.github.io/salmonella-serovar-wiki/ accessed on 15 January 2026.

**Table 2:** Summary of comparative of studies on *Salmonella enterica* biofilm formation across serovars under diverse surface types, environmental conditions, and assay methods.

*Salmonella* Serovar	Surface Type/Temperature	Biofilm Assay	Findings	References
Schwarzengrund, Heidelberg, Newport, Braenderup, Hadar, Infantis, Kentucky, Thompson, Typhimurium, Enteritidis (total 20 isolates)	Plastic vs. stainless steel; 25 °C and 15 °C	Crystal violet microtiter biofilm assay	Schwarzengrund—strong biofilms; Braenderup—strong/moderate biofilms; Enteritidis—moderate biofilms; Infantis—moderate biofilms; Typhimurium—moderate biofilms; Thompson—moderate biofilms; Hadar—moderate/weak biofilms; Kentucky—weak biofilms; Heidelberg—weak biofilms; Newport—weak biofilms; plastic surfaces generally supported stronger biofilm than stainless steel, especially at 15 °C.	[37]
Agona, Montevideo, Senftenberg, Typhimurium (total 111 isolates)	Polystyrene microtiter plates and liquid-air interface; ~20 °C	Crystal violet microtiter biofilm assay; pellicle formation assay	Agona—strong biofilms; Montevideo—strong biofilms; Senftenberg—moderate biofilms; Typhimurium—weak biofilms; persistent factory serovars (Agona and Montevideo) showed greater biofilm formation than non-persistent serovars, suggesting biofilm ability contributes to environmental persistence.	[39]
Typhimurium, Enteritidis, Newport, Heidelberg, Agona, Montevideo, Infantis, Senftenberg, Derby (total 60 isolates)	Different environmental conditions: pH 3.8–7.0; NaCl 0.5–8%; temperature 4–37 °C	Crystal violet biofilm assays under factorial environmental conditions	Typhimurium—variable biofilms; Enteritidis—variable biofilms; Newport—variable biofilms; Heidelberg—variable biofilms; Agona—strong biofilms in some strains; Montevideo—moderate/variable biofilms; Infantis—variable biofilms; Senftenberg—moderate biofilms; Derby—limited data; biofilms formation strongly influenced by environmental factors (pH, NaCl, temperature) rather than serovar identity.	[40]
Typhimurium (sequence types ST19 and ST313 clinical isolates) (total 16 isolates)	Polystyrene microtiter plates; glass flow cells; polycarbonate membranes; 25 °C, 28 °C, and 37 °C	Crystal violet microtiter assay; Congo-red RDAR * morphotype assay; pellicle formation assay; continuous-flow biofilm system	Typhimurium ST19—strong biofilms; Typhimurium ST313—weak/poor biofilms; ST19 isolates produced RDAR morphotype and thick biofilms in flow-cell systems, whereas ST313 isolates showed weak attachment and minimal biofilm formation; ST19 strains also survived desiccation and disinfectant treatment better than ST313 strains.	[38]
Typhimurium, Enteritidis, and other *Salmonella* spp. (total 95 isolates)	Polystyrene 96-well microtiter plates; 30 °C and 37 °C	Crystal violet microtiter plate biofilm assay	Typhimurium—strong biofilms; Enteritidis—moderate/strong biofilms; other *Salmonella* spp.—moderate/strong biofilms; most isolates showed stronger biofilm formation at 37 °C, with 78.5% of Typhimurium isolates producing strong biofilms, while Enteritidis showed ~41.7% strong biofilm at 37 °C; strong biofilm formation was correlated with multidrug resistance (MDR).	[41]
Heidelberg, Muenchen, Schwarzengrund, Lagos, Labadi, Anatum, Give, Derby, Mbandaka, 0:a,5:i:-, 0:-:r:2, 0:4,5:b:- (total 37 isolates)	Polystyrene microtiter plates; 35 °C incubation for 24 h	Crystal violet microtiter plate biofilm assay	Schwarzengrund—moderate biofilms; Muenchen—moderate/weak biofilms; Derby—moderate biofilms; Mbandaka—moderate biofilms; Heidelberg—weak or non-biofilms; Lagos—weak biofilms; Labadi—weak biofilms; Anatum—weak biofilms; Give—weak biofilms; 0:a,5:i:-—weak biofilms; 0:-:r:2—non-biofilms; 0:4,5:b:-—weak biofilms. Overall, 65% of isolates produced biofilm, mostly weak to moderate producers, and multidrug-resistant isolates tended to form weaker biofilms.	[42]
Enteritidis (total 95 isolates; 51 from poultry and 44 from human feces)	Congo red agar medium; 28 °C	RDAR */BDAR ^#^/PDAR ^¥^ colony morphotype analysis; gene expression analysis of biofilm-related genes (*adrA*, *csgD*, *luxS*, *sdiA*)	Enteritidis—strong/weak biofilms; RDAR morphotype (curli + cellulose) observed in ~39% of poultry isolates and ~34% of human isolates; BDAR and PDAR morphotypes also detected; strong biofilm formation associated with increased expression of *adrA* and *csgD* genes, whereas some strains showed weak biofilm formation despite the expression of quorum sensing-associated genes.	[43]

* RDAR—red, dry, and rough (curli + cellulose); ^#^ BDAR—brown, dry, and rough (curli only); ^¥^ PDAR—pink, dry, and rough (cellulose only).

**Table 3:** Biofilm-associated gene expression differences among *Salmonella* serovars by host specificity (broad host range, host-adapted, and host-restricted).

Category	Genes	Gene Functions	Biofilm Implications	Genomic/Expression Differences Across Serovars	References
**Core biofilm genes**	*csgD*	Master regulator of biofilm genes	Controls curli and cellulose production	Broad-host-range serovars: intact and functional Host-adapted serovars: expression depends on niche-specific signals Host-restricted serovars: mutated with premature stop codon, producing truncated protein	[56,60,61]
	*csgBAC*, *csgEFG*	Curli fimbriae (adhesion, matrix formation)	Essential for attachment and RDAR * phenotype	Broad-host-range serovars: intact and functional Host-adapted serovars: expression depends on niche-specific signals Host-restricted serovars: reduced or absent expression due to regulatory (*csgD*) disruption	[60,61,62,63]
	*bcs* operon	Cellulose synthesis (EPS matrix)	Structural stability of biofilm	Broad-host-range serovars: intact and functional Host-adapted serovars: expression may vary under stress/invasive conditions Host-restricted serovars: disrupted gene expression	[64,65,66]
	*adrA*	c-di-GMP synthesis → activates cellulose	Promotes matrix production	Broad-host-range serovars: intact and functional Host-adapted serovars: expression may vary under stress/invasive conditions Host-restricted serovars: reduced expression due to *csgD* disruption	[62,67,68]
	*rpoS*	Global stress regulator	Links stress tolerance with biofilm formation	Broad-host-range serovars: functional RpoS-CsgD pathway supports biofilm formation Host-adapted serovars: expression depends on environmental signals Host-restricted serovars: variable biofilm response due to *rpoS* mutations	[69,70]
**Accessory biofilm genes**	*bapA*	CsgD-regulated adhesin; contributes to pellicle formation	Enhances surface attachment and biofilm stability	Broad-host-range serovars: intact and functional Host-adapted serovars: present but less well characterized Host-restricted serovars: reduced expression due to *csgD* disruption	[19,71]
	*fim* (Type 1 fimbriae), *lpf* (long polar fimbriae)	Surface attachment	Strong adhesion and colonization	Broad-host-range serovars: intact and functional Host-adapted serovars: niche specific expression Host-restricted serovars: frequently pseudogenized and reduced expression	[72,73,74]
	*pef* (IncF plasmid encoded), *ipf* and *klf* (pESI megaplasmid encoded)	Adhesion systems that enhance persistence, colonization, and niche adaptation	Enhances persistence and colonization	Broad-host-range serovars: intact and functional Host-adapted serovars: niche specific expression Host-restricted serovars: often lack these plasmids entirely	[11,75]

* RDAR—red, dry, and rough (curli + cellulose).

## Data Availability

No new data were created or analyzed in this study. Data sharing is not applicable to this article.

## Abbreviations

The following abbreviations are used in this manuscript:

AMR	Antimicrobial resistance
MDR	Multidrug resistance
ESBL	Extended-spectrum β-lactamase
EPS	Extracellular polymeric substance
ST	Sequence type
RDAR	Red, dry, and rough
BDAR	Brown, dry, and rough
PDAR	Pink, dry, and rough
MGEs	Mobile genetic elements
LC-MS	Liquid chromatography and mass-spectrometry
ML	Machine learning
